# Surface Engineering of Cu-Zn Alloys via Femtosecond Laser Processing

**DOI:** 10.3390/mi17070862

**Published:** 2026-07-21

**Authors:** Serguei P. Murzin

**Affiliations:** 1 TU Wien, Karlsplatz 13, 1040 Vienna, Austria; serguei.murzin@tuwien.ac.at; 2 Samara National Research University, Moskovskoe Shosse 34, Samara 443086, Russia

**Keywords:** surface engineering, Cu–Zn alloys, femtosecond laser processing, laser-induced plasma, Zn redistribution, plasma-assisted oxidation, ZnO formation

## Abstract

This review presents a comprehensive analysis of the physicochemical mechanisms underlying surface engineering of Cu–Zn alloys through femtosecond laser processing. It focuses on the coupled evolution of laser-induced plasma formation, selective ablation, nonequilibrium Zn redistribution, and plasma-assisted oxidation. Experimental and theoretical evidence indicates that ZnO formation cannot be explained by gas-phase reactions or surface oxidation alone, but results from the interplay of plasma processes, diffusion-controlled Zn redistribution, and heterogeneous oxidation under nonequilibrium conditions. A plasma–surface–diffusion framework is employed to interpret these coupled processes, linking selective Zn redistribution, plasma-assisted oxidation, and ZnO formation within the laser-modified surface layer. The review discusses ZnO evolution, including the influence of supersaturation, defects, and relaxation times, and highlights the effects of laser-induced structuring on reaction kinetics, energy redistribution, and mass transport. Comparison with plasma-assisted and gas-phase ZnO synthesis demonstrates common kinetic stages while emphasizing the localized and transient nature of femtosecond laser processing. This integrated interpretation provides a mechanistic basis for controlled ZnO formation. Overall, ZnO formation on Cu–Zn alloys is interpreted through a multiscale physicochemical approach integrating nonequilibrium electron excitation, plasma evolution, Zn redistribution, heterogeneous oxidation, and surface morphology, providing a framework for the rational optimization of laser-functionalized brass surfaces.

## 1. Introduction

Surface engineering of Cu–Zn alloys using femtosecond laser processing has attracted increasing attention as an effective approach for controlling surface composition and morphology at the micro- and nanoscale. In conventional industrial applications, the selective mobility of zinc in brass is commonly associated with dezincification and deterioration of surface properties [1]. However, studies of ultrashort laser–matter interactions indicate that this intrinsic behavior can also be utilized during controlled surface modification. Under femtosecond laser irradiation, zinc redistribution may contribute to the in situ formation of ZnO-containing surface layers rather than simply representing a degradation process. This possibility provides a route for transforming an undesired diffusion phenomenon into a controllable pathway for surface phase formation.

A characteristic feature of Cu–Zn systems is the relatively high mobility of zinc atoms within the copper matrix. Experimental and modeling studies have demonstrated that zinc diffusion plays an important role in determining the chemical composition and surface evolution of these alloys [2]. In many conventional applications, this behavior is considered undesirable because it may result in dezincification and deterioration of mechanical or functional properties. Nevertheless, controlled femtosecond laser irradiation provides an opportunity to redirect zinc redistribution toward the formation of surface oxide phases. In this case, zinc migration becomes an active component of the modification process rather than only a degradation pathway [3]. Similar laser-induced redistribution effects have been reported in multicomponent metallic systems, where ultrashort pulse irradiation produces rapid mass transport and phase transformation under highly localized energy deposition conditions [4].

Among the oxide phases that can form during laser treatment of Cu–Zn alloys, zinc oxide is of particular interest due to its semiconducting, piezoelectric [5], antibacterial [6], and photocatalytic properties, which enable its application in various technological fields [7]. ZnO is also one of the most extensively studied oxide semiconductors, and its structural and functional characteristics have been widely investigated [8]. Previous studies have demonstrated that plasma-assisted synthesis conditions can significantly affect the structural and functional properties of ZnO films [9]. Conventionally, ZnO coatings on brass are fabricated using multistep deposition approaches; however, femtosecond laser processing provides an alternative route for local in situ oxide formation directly on the alloy surface. Several studies have shown that ultrashort laser irradiation can initiate photochemical reactions resulting in ZnO crystallization without the use of masks or additional deposition procedures [10,11]. These findings demonstrate the potential of femtosecond laser processing for localized formation of ZnO-containing surface phases. General aspects of laser processing of metallic materials, including fundamental mechanisms and practical applications, have been reviewed elsewhere [12].

Femtosecond laser irradiation produces highly transient conditions involving ultrafast electronic excitation, rapid energy redistribution, and strong spatial and temporal gradients of deposited energy [13]. Compared with conventional thermal processing methods, such irradiation enables localized modification of metallic surfaces while limiting the extent of heat-affected regions [14]. In addition to chemical modification, femtosecond laser processing provides effective control over surface morphology through the formation of laser-induced periodic surface structures (LIPSS) [15]. The resulting morphology depends strongly on irradiation parameters, including fluence, pulse number, and scanning conditions [16]. Different types of laser-induced surface structures, including LIPSS, microgrooves, and porous features, as well as their potential applications, have been summarized in previous studies [17].

The evolution of laser-induced surface structures, including the transition from shallow LIPSS formed directly on the surface to deeper periodic structures located within laser-ablated microgrooves, has been investigated in detail in Ref. [18]. Approaches for improving large-area surface uniformity through dynamic feedback control have also been reported [19]. Furthermore, implanted-marker methodologies have provided additional insight into the contributions of electromagnetic and hydrodynamic mechanisms involved in surface pattern formation [20]. Despite these advances in understanding laser-induced surface structuring, the processes responsible for Zn redistribution, plasma formation, oxide growth, and morphology evolution during femtosecond laser treatment of brass have not yet been fully correlated. A comprehensive description of ZnO formation on brass surfaces requires consideration of the relationships between elemental redistribution, oxidation reactions, and the development of laser-induced surface morphology.

The aim of this review is to clarify the mechanisms responsible for femtosecond laser-induced ZnO formation on brass surfaces and to analyze the relationships between zinc redistribution, oxidation processes, and surface morphology evolution. Particular attention is paid to the sequence of processes involved in ZnO formation, including laser-induced modification of the alloy surface, zinc migration, plasma-assisted oxidation, and oxide nucleation. Based on experimental observations reported in the literature and established concepts of ultrafast laser–matter interaction, this review discusses the factors governing the formation of ZnO-containing surface structures and the challenges associated with controlling their composition and morphology. The analysis provides insight into the key processes determining laser-assisted in situ oxide formation and the resulting surface characteristics of modified brass alloys.

## 2. Microphysics of Zinc Redistribution Under Femtosecond Laser Irradiation

Femtosecond laser irradiation drives the Cu–Zn system into a highly transient state, where the absorbed laser energy is initially deposited into the electronic subsystem and subsequently transferred to the crystal lattice on picosecond timescales. The delayed electron–phonon energy exchange produces steep gradients of energy density, pressure, and chemical potential within the near-surface region, creating favorable conditions for rapid material redistribution. Under these conditions, zinc redistribution cannot be described solely by conventional diffusion processes, since several transport mechanisms may contribute during laser irradiation and the subsequent relaxation stage.

The interaction of ultrashort laser pulses with metallic materials is generally described as a sequence of electronic excitation, electron–phonon relaxation, melting, phase transformation, and material removal. According to Ref. [21], electronic excitation plays an important role in the transition from energy absorption to structural modification of the material. The subsequent evolution of the irradiated region is governed by the interplay between electronic heat transport, lattice relaxation, and hydrodynamic motion within the transient molten layer. Ultrafast electronic thermal conductivity enables energy transport beyond the optical absorption depth, thereby affecting both the spatial extent of structural modification and the redistribution of alloy components [21,22]. Electron–phonon coupling exhibits strong temperature dependence, which influences relaxation times and the depth of energy deposition [23]. More advanced descriptions consider deviations from the classical two-temperature model associated with the breakdown of local equilibrium, temperature-dependent electron–phonon coupling, non-uniform energy exchange, and the influence of electronic excitation on phase evolution [24,25,26,27].

These processes lead to different responses of copper and zinc due to differences in their electronic structures, bonding characteristics, and atomic mobility. Tracer-diffusion measurements in α-Cu_64_Zn_36_ demonstrate that zinc possesses considerably higher diffusional mobility than copper [28]. Therefore, even in the absence of laser irradiation, Cu–Zn alloys exhibit an intrinsic tendency toward selective zinc redistribution. This difference becomes more pronounced under transient laser-induced conditions, where rapid energy deposition modifies atomic mobility and defect formation. The lower bonding energy and higher volatility of zinc compared with copper further promote its preferential participation in mass transport processes within the laser-affected region.

With increasing energy density, a transient molten layer is formed, in which atomic mobility and diffusion coefficients increase by several orders of magnitude compared with the solid state. At this stage, material redistribution is controlled not only by atomic diffusion but also by melt convection, recoil-pressure-driven flow, and cumulative effects associated with multiple laser pulses. In particular, Ref. [29] demonstrated that GHz-burst femtosecond irradiation can efficiently remove molten material through accumulated recoil pressure and changes in melt hydrodynamics. Although these experiments were not performed on brass, the identified mechanisms are relevant for interpreting mass transport in Cu–Zn alloys subjected to multipulse femtosecond irradiation.

At higher energy densities, the irradiated material may enter a phase-explosion regime, where liquid and vapor phases coexist and rapid decomposition of the molten region occurs. Because zinc has lower bonding energy and higher volatility than copper [28], it is expected to participate preferentially in evaporation and redistribution processes. This selective behavior enhances chemical separation between alloy components and may contribute to the formation of locally heterogeneous regions containing different concentrations of Zn and Cu. During the ablation stage, the removed material forms a multiphase plume containing atoms, ions, clusters, and droplets. Within the expanding plasma plume, additional redistribution of ablated species occurs due to differences in atomic mass, ionization behavior, and transport characteristics. Experimental studies of femtosecond laser processing of Cu–Zn alloys indicate that nonequilibrium condensation processes can preserve part of the compositional heterogeneity generated during ablation and plume evolution [30]. Therefore, the plasma stage should be considered not only as a transport mechanism for ablated material but also as a factor influencing the final distribution of alloy components.

Chemical reactions occurring within the laser-induced plume can further modify the composition of Zn-containing species during plume expansion [31]. In addition, plasma expansion and shielding effects may influence the interaction of subsequent laser pulses by modifying energy deposition and material removal conditions. Such effects, including complex plume evolution and energy redistribution, have been investigated using Lagrangian modeling approaches [32]. During subsequent cooling, aerosol condensation leads to the formation of submicron particles while partially preserving the elemental distribution generated during ablation [33]. Additional compositional variations may arise from fragmentation, phase separation, and condensation processes occurring within the ejected material volume [34]. Experimental studies of laser-treated zinc-containing materials further demonstrate that defect structure, local chemical composition, and surface morphology formed during irradiation strongly influence subsequent oxidation processes and oxide phase development [35,36].

Overall, zinc redistribution during femtosecond laser processing results from the combined contribution of several transport mechanisms, including solid-state diffusion, transient liquid-phase transport, selective evaporation, and plasma-mediated redistribution. The relative importance of these mechanisms depends on local energy deposition, irradiation parameters, and the temporal evolution of the laser-affected region.

Consequently, zinc enrichment at the surface and subsequent ZnO formation should be considered as sequential stages of a single laser-induced modification process. The femtosecond laser pulse initiates selective zinc redistribution and rapid modification of the alloy surface; transient melting and ablation provide additional pathways for mass transport; plasma evolution affects the redistribution of ejected species; and subsequent oxidation stabilizes zinc in the form of ZnO-containing surface phases. The resulting surface composition is therefore determined by the combined effects of atomic mobility, transient transport phenomena, plasma-assisted processes, and oxidation reactions occurring during and after laser irradiation.

## 3. Mesoscale: Structural Evolution of the Surface Under Femtosecond Laser Irradiation

**Figure 1:**
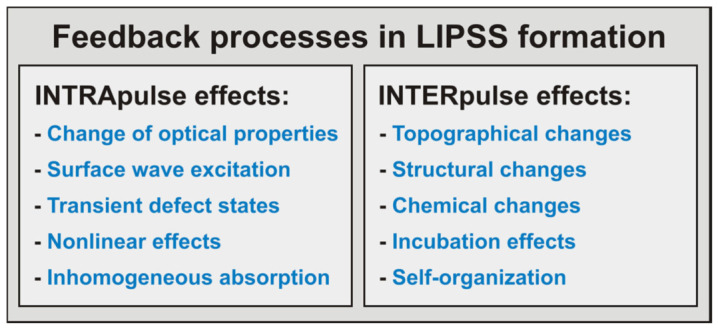
Feedback mechanisms associated with the formation of laser-induced periodic surface structures (LIPSS), including intrapulse and interpulse effects during surface morphology evolution [37].

The formation of periodic surface structures during femtosecond laser irradiation, including laser-induced periodic surface structures (LIPSS) and structures produced by direct laser interference patterning (DLIP), plays an important role in controlling the interaction between the laser radiation and the material surface. Rather than being only a consequence of material removal, these structures modify the optical response and energy deposition conditions during subsequent irradiation. They originate from interference between the incident electromagnetic field, scattered radiation, and surface electromagnetic modes, resulting in a spatially modulated distribution of absorbed energy. LIPSS are widely considered a characteristic type of laser-induced surface modification formed through feedback between electromagnetic excitation and progressive surface evolution under ultrashort pulse irradiation [37]. Figure 1 illustrates the feedback mechanisms involved in LIPSS formation, including processes occurring during individual pulses and during repeated irradiation, where changes in surface morphology influence subsequent laser–surface interaction.

**Figure 2:**
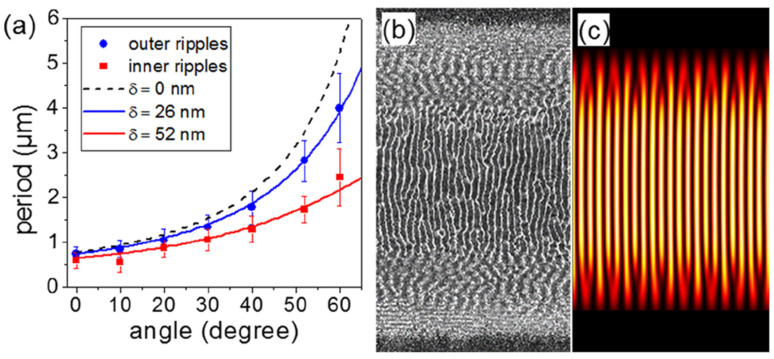
Formation of LIPSS on metallic surfaces: (**a**) dependence of the structural period on copper as a function of the angle of incidence, comparing experimental data with a plasmonic model accounting for surface roughness (solid lines) and a model for an idealized smooth surface (dashed lines); (**b**) SEM image of structures formed at an incidence angle of 52° and an effective pulse number of *N_eff_* = 1000; (**c**) intensity distribution of surface plasmon polaritons on an inhomogeneous surface [38].

A key mechanism responsible for the initial formation of periodic structures is the excitation of surface plasmon polaritons (SPPs) and their interference with the incident electromagnetic field on rough metallic surfaces [38] (Figure 2). Localization of SPPs at surface defects and step edges can promote the formation of initial periodic modulation even during single-pulse irradiation when sufficient surface irregularities are present [39]. These electromagnetic effects define the initial spatial distribution of absorbed energy and provide the basis for subsequent morphological evolution during multipulse exposure [40]. For Cu–Zn alloys, however, the applicability of plasmonic models developed for pure metals requires additional consideration. During femtosecond laser irradiation, selective zinc evaporation, oxidation, and compositional redistribution modify the local optical properties of the surface. Therefore, alloy composition should be taken into account when analyzing energy localization mechanisms in brass.

**Figure 3:**
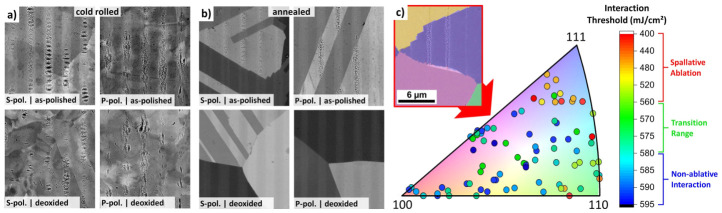
Influence of the crystallographic state of copper on ablation behavior and incubation effects during USP-DLIP processing: (**a**) deformed Cu in the initial and deoxidized states; (**b**) undeformed Cu under analogous conditions; (**c**) annealed samples, where SEM and electron backscatter diffraction (EBSD) analysis enable correlation between surface morphology and local crystallographic orientation [42].

Experimental studies demonstrate that initial LIPSS features may develop after a single laser pulse in regions containing surface defects or structural heterogeneities [41]. Fully developed periodic structures are generally associated with multipulse irradiation, where incubation effects and cumulative redistribution of laser-modified material become significant [42]. During repeated exposure, each subsequent pulse interacts with a surface that has already experienced melting, resolidification, and local compositional modification. As a result, periodic surface modulation gradually increases through redistribution of molten material from regions of higher absorbed energy toward adjacent regions with lower energy deposition, leading to the formation of stable ridge-like structures. Electron backscatter diffraction (EBSD) investigations further demonstrate the influence of crystallographic orientation on the resulting morphology, while DLIP studies emphasize the role of the initial surface state in determining the structuring response [42] (Figure 3).

For crystalline materials, two-dimensional LIPSS formation has also been demonstrated under GHz-burst irradiation conditions, where high repetition rates produce characteristic electronic excitation and thermal accumulation effects [43]. Current models describe LIPSS development as the result of spatially modulated electromagnetic energy deposition followed by thermocapillary flow, melt redistribution, and surface relaxation processes [44,45]. As the structures evolve during multipulse irradiation, changes in surface topography modify local absorption conditions and redistribute the deposited energy, providing a feedback mechanism for further structural development. In contrast, DLIP generates periodic surface patterns through controlled interference of coherent laser beams and therefore provides a deterministic distribution of energy deposition [46]. The combination of DLIP and LIPSS can produce hybrid structures in which externally imposed periodicity interacts with laser-induced surface modulation, resulting in hierarchical surface morphologies [47]. Depending on the irradiation regime and material properties, these mechanisms may contribute simultaneously to the final surface architecture [48].

Studies of DLIP processing on nonmetallic materials demonstrate the applicability of this technique for periodic surface structuring across different classes of materials [49]. Practical implementations confirm the reproducibility of DLIP for surface functionalization [50], including the fabrication of high-aspect-ratio periodic structures over large areas while maintaining geometric stability [51]. At the mesoscale, the resulting surface morphology affects heat transfer, mass transport, and interfacial interactions. Hierarchical structures increase the effective surface area and modify local thermal conditions within micro- and nanocavities. Experimental studies have shown that laser-generated hierarchical morphologies can influence the redistribution of local heat and mass fluxes, including on curved surfaces [52]. Such structures also affect adhesion and interfacial transport processes [53] and are widely used to control wettability and capillary-driven transport phenomena [54].

DLIP further enables the fabrication of gradient periodic structures with controlled variation in the structural period over extended surface regions [55]. As a scalable processing approach, DLIP has been applied for the production of functional periodic surfaces with controlled topography [56], and recent developments demonstrate its compatibility with large-area manufacturing while preserving micro- and nanoscale structural precision [57]. In addition to modifying heat and mass transfer, surface morphology influences the evolution of the laser-induced plasma plume. Pores, grooves, and depressions can locally reduce the efficiency of material removal, increasing the residence time of ablation products near the surface and affecting cooling and condensation processes. Therefore, surface relief contributes to the coupling between laser-generated morphology and plasma-assisted redistribution of ablated species.

Transitions between different LIPSS regimes, including low- and high-spatial-frequency structures, are associated with variations in the balance between electronic excitation, energy relaxation, and lattice response, resulting in changes in characteristic structural periods [58]. Laser texturing provides an effective method for tailoring surface properties, including wettability, optical response, and heat and mass transfer, through controlled formation of micro- and nanostructured morphologies [59]. Consequently, surface morphology should not be considered only as a final product of laser irradiation but also as an important factor affecting subsequent laser–material interaction, local energy distribution, and material transport. In the context of ZnO formation on brass, laser-generated structures may influence oxidation pathways by modifying surface area, defect distribution, and local reaction conditions. Recent studies further indicate that structured surfaces can affect interfacial processes such as phase-transition behavior and localization of oxide formation sites [60].

## 4. Mechanisms Governing ZnO Formation During Femtosecond Laser Processing

ZnO formation during femtosecond laser processing of Cu–Zn alloys results from a sequence of interconnected processes involving zinc redistribution, plasma-assisted oxidation, transport of reactive species, and oxide nucleation. In this system, laser irradiation initiates not only ablation but also selective redistribution of alloy components, leading to local enrichment of zinc within the modified region. At the same time, the laser-induced plasma provides oxygen-containing reactive species and facilitates their transport toward the alloy surface. Therefore, ZnO formation is determined by the interaction between the evolving composition of the Cu–Zn substrate, plasma chemistry, and oxidation reactions occurring during and after laser irradiation. Recent multiscale approaches combining descriptions of elementary reactions with continuum transport models emphasize the importance of considering chemical kinetics, mass transport, and elemental redistribution together when analyzing laser-induced phase transformations [61].

The expansion of the laser-induced plasma plume under atmospheric conditions results in an increase in the concentration of reactive species near the irradiated surface and promotes localized chemical reactions. Within this region, ionization, dissociation of oxygen-containing molecules, and formation of Zn–O-containing species may occur simultaneously with the redistribution of metallic components. The confinement of the plasma plume also affects the residence time of reactive species and the probability of their interaction with the modified surface. At the same time, selective redistribution of zinc relative to copper in the near-surface region produces local variations in chemical composition, creating areas where oxide formation becomes kinetically favorable.

The mechanism of ZnO formation cannot be attributed exclusively either to gas-phase reactions within the plasma plume or to direct oxidation of the solid surface. The laser-induced plasma acts as a source and transport medium for reactive oxygen-containing species, whereas the incorporation of zinc into the oxide phase occurs predominantly at the plasma–surface interface. This region represents a transient reaction zone where increased zinc concentration, reactive species, structural defects, and local thermal modification coexist. Such an interpretation is consistent with models of heterogeneous oxidation, where oxidation kinetics are determined by the transport of reactive species to the interface and their subsequent interaction with the substrate surface [62,63].

A specific feature of Cu–Zn alloys is that the irradiated substrate itself serves as the source of zinc during laser processing. Selective zinc evaporation, diffusion, and redistribution within the laser-affected region lead to the formation of transient zinc-enriched areas. These regions experience partial melting, rapid solidification, defect generation, and structural rearrangement, which may increase the chemical activity of the surface and facilitate ZnO nucleation. Similar behavior has been observed in plasma-assisted ZnO synthesis, where oxide formation is controlled by the interaction of active plasma species with the substrate, followed by nucleation and growth of oxide structures [64].

Accordingly, ZnO formation during femtosecond laser treatment involves three closely related stages: generation and transport of reactive oxygen-containing species, redistribution of zinc within the Cu–Zn system, and heterogeneous oxidation at the modified surface. The plasma primarily determines the availability and transport of reactive species, while the alloy provides the zinc required for oxide formation. Experimental studies of plasma-assisted ZnO synthesis demonstrate that plasma conditions strongly influence oxide morphology, crystallinity, and phase composition [65]. These effects become particularly important when zinc originates directly from the alloy matrix, because the local concentration and distribution of zinc determine the kinetics of oxide formation. Depending on the concentration of Zn-containing species and oxidation conditions, plasma-assisted processes can produce ZnO structures ranging from compact layers to nanostructured morphologies, including nanorods [66].

Theoretical descriptions of reactive dynamics indicate that ZnO nucleation in plasma-assisted environments is sensitive to local temperature, species concentration, collision processes, and the supply of zinc from the laser-modified Cu–Zn region [67]. Because these parameters vary spatially during laser irradiation, oxide formation proceeds non-uniformly across the processed surface, resulting in differences in nucleation density and crystal growth rate. Thus, the spatial distribution of ZnO is determined not only by chemical reactions but also by local variations in mass transport and thermal conditions.

The stabilization of the ZnO phase depends on the competition between plasma cooling, oxidation kinetics, and the transport of zinc-containing species from the laser-affected alloy region. An important factor is the relationship between the characteristic relaxation time of the modified system and the rate of oxidation and ZnO nucleation. If oxidation and incorporation of zinc into the oxide phase occur before rapid cooling and removal of reactive species, stable oxide nuclei can be preserved and subsequently developed into crystalline structures. In situ studies of ZnO growth demonstrate that the initial nucleation stage is followed by nanoparticle growth under conditions of local supersaturation and favorable temperature gradients [68]. Investigations of low-temperature ZnO formation further emphasize the importance of surface diffusion and lattice reconstruction for the stabilization of emerging nanostructures [69]. These observations indicate that ZnO growth cannot be considered solely as a gas-phase process, since reactions and structural transformations at the evolving alloy surface also contribute to oxide formation.

A similar kinetic behavior is observed in other plasma-assisted oxide synthesis methods, where the final structure depends on the balance between the flux of reactive species and their incorporation into the growing phase [70]. Plasma-assisted ZnO synthesis generally involves sequential stages of nucleation, growth, and crystallization. The transition from amorphous to crystalline ZnO is controlled by the local energy state of the system and the availability of reactive species [71]. Surface defects, including oxygen vacancies, may further influence adsorption, diffusion, and subsequent crystal growth processes [72]. Depending on plasma parameters and zinc availability, plasma-based synthesis methods are capable of producing ZnO structures with a wide range of morphologies, from dense films to hierarchical nanostructures [73].

The role of reactive species generated during plasma formation extends beyond the duration of the laser pulse itself. These species may continue to participate in oxidation reactions during plume cooling and subsequent interaction with the modified surface [74]. Similar sequences involving activation, nucleation, growth, and structural stabilization have been reported in electrochemical deposition systems [75], arc-plasma synthesis, and low-temperature oxidation processes [76,77]. Kinetic Monte Carlo simulations of ZnO growth further demonstrate that surface diffusion, incorporation probability, and local mass transport strongly affect the evolution of oxide structures [78]. Experimental investigations of hierarchical ZnO morphologies confirm that the final structural organization is determined by the combined influence of reaction kinetics, defect structure, and transport processes [79].

The formation of ZnO during femtosecond laser processing occurs within a limited processing window determined by the balance between energy deposition, zinc redistribution, plasma generation, and oxidation kinetics. At relatively low laser fluence, surface restructuring dominates, while chemical modification remains limited despite possible zinc enrichment near the surface. Under intermediate fluence conditions, material redistribution and oxidation can proceed simultaneously, providing favorable conditions for ZnO formation. In this regime, sufficient zinc is supplied from the alloy while excessive material removal is avoided, increasing the probability of oxide nucleation and stabilization. At higher fluence, intense ablation and rapid ejection of material reduce the lifetime of zinc-enriched regions and may suppress controlled ZnO growth due to unstable transport conditions and excessive removal of reactive species.

In addition to chemical composition, the morphology generated during femtosecond laser processing strongly influences subsequent ZnO formation. Surface roughness, hierarchical structures, defect density, and local compositional variations modify the available reaction area, adsorption sites, and transport pathways for reactive species. Therefore, the structural evolution described in Section 3 directly affects oxidation behavior and the final morphology of ZnO-containing layers. Experimental studies of laser-processed zinc-containing materials demonstrate that defect structure, local chemical heterogeneity, and surface morphology are important factors controlling oxide formation and stabilization [80].

Comparable relationships between processing conditions and oxide structure have been reported for plasma-electrolytic oxidation and radio-frequency plasma synthesis. Variations in plasma parameters affect crystallinity, defect concentration, and structural organization of ZnO despite differences in activation mechanisms [81,82]. These observations further demonstrate the sensitivity of ZnO formation to local energy input, reactive species availability, and transport conditions.

Overall, ZnO formation during femtosecond laser processing of brass can be described as the result of selective zinc redistribution, plasma-assisted oxidation, and subsequent oxide nucleation and growth. The process is not simply a secondary consequence of material removal but arises from the interaction between laser-induced modification of the alloy, transport of reactive species, and oxidation reactions at the evolving surface. Understanding the relationship between these processes provides a basis for controlling the composition, morphology, and functional characteristics of ZnO-containing surface layers formed on Cu–Zn alloys.

## 5. Comprehensive Characterization Strategy for Functional Surface Engineering

Reliable characterization of femtosecond laser-induced ZnO formation on Cu–Zn alloys requires a combination of complementary analytical techniques capable of resolving elemental redistribution, chemical-state evolution, and morphological transformation. The combined application of femtosecond laser ablation spark-induced breakdown spectroscopy (fs-LA-SIBS), X-ray photoelectron spectroscopy (XPS), and scanning/transmission electron microscopy (SEM/TEM) provides a multiscale characterization framework that links chemical processes with the resulting surface architecture. Such an approach is particularly important for nonequilibrium laser-modified systems, where phase formation is accompanied by strong spatial heterogeneity and transient compositional gradients.

Femtosecond LA-SIBS provides high sensitivity to local elemental composition and enables quantitative analysis of complex multicomponent alloys, including Cu–Zn systems, while reducing thermal effects compared with conventional laser ablation approaches [83]. The application of ultrashort laser pulses for elemental analysis and thin-film studies has demonstrated improved separation of signals associated with different material states and phases [84]. In multicomponent systems, fs-LA-SIBS extends the analytical capability for localized chemical mapping [85], allowing the redistribution of zinc within the Cu–Zn matrix and its subsequent participation in oxidation processes to be investigated [86]. This capability is particularly important for identifying chemical gradients generated during laser irradiation and correlating them with the formation of functional oxide structures.

Despite the high sensitivity of spectroscopic methods, interpretation of the obtained signals may be complicated by spectral overlap and difficulties in assigning individual components to specific chemical states. For ZnO-containing systems, this limitation is especially relevant for the O 1s region, where contributions from lattice oxygen, hydroxyl groups, and defect-related oxygen states may partially overlap [87]. Such uncertainties are common for oxide materials with closely spaced electronic states and require additional structural and chemical information for reliable phase identification.

XPS remains one of the primary techniques for determining the chemical state of surface species; however, its interpretation requires careful consideration of charging effects, surface roughness, and limited probing depth [88]. In laser-processed Cu–Zn systems, these limitations become particularly important because the surface may contain metallic regions, partially oxidized zones, and ZnO-containing layers formed during nonequilibrium processing. The measured XPS response represents an averaged signal from the near-surface region and therefore does not directly provide the complete vertical distribution of different phases or chemical states.

Several advanced XPS approaches can improve the analysis of such heterogeneous systems. Angle-resolved XPS (ARXPS) enables nondestructive evaluation of chemical-state distributions as a function of depth [89], while in situ XPS provides information on surface evolution during oxidation and reaction processes [90]. Depth-sensitive approaches based on emission-angle variation allow reconstruction of thin oxide layers [91], and self-consistent spectral fitting procedures improve the reliability of quantitative interpretation in complex oxide systems [92]. Nevertheless, ZnO identification should always consider the fact that the measured spectra reflect both the actual chemical state of the material and the methodological limitations of the analysis procedure [93]. Therefore, XPS data should be interpreted together with complementary structural and compositional information.

Correlation between independent characterization methods provides an additional level of reliability for phase identification and structural analysis. Combined spectroscopic and microscopic investigations improve the estimation of oxide thickness, chemical uniformity, and morphology evolution in laser-modified metallic systems [94]. In this context, SEM and TEM provide not only information about surface morphology but also indirect evidence of phase formation through diffraction analysis, contrast variation, and nanoscale elemental mapping. Such approaches are particularly important for distinguishing metallic zinc from ZnO in ultrathin oxide layers, where their signals may partially overlap and conventional surface-sensitive techniques may not fully resolve nanoscale compositional heterogeneity.

Therefore, reliable verification of ZnO formation during femtosecond laser processing requires an integrated characterization strategy combining fs-LA-SIBS, XPS (including ARXPS and depth profiling), and electron microscopy. The correlation of elemental distribution, chemical-state information, and nanoscale morphology enables separation of contributions from the metallic substrate, intermediate oxidation states, and the final ZnO-containing surface layer. Such a comprehensive approach is essential for establishing reliable relationships between laser-processing parameters, oxide formation mechanisms, and the resulting functional properties of engineered Cu–Zn surfaces.

## 6. Subsurface Microstructure Modification and Mechanical Properties of Brass

Femtosecond laser modification of Cu–Zn alloys involves a sequence of strongly coupled nonequilibrium processes occurring over a wide range of temporal (from femtoseconds to nanoseconds) and spatial (from nanometers to micrometers) scales. The initial interaction stage includes photoionization, ultrafast excitation of the electronic subsystem, electron–phonon energy transfer, and subsequent development of a transient molten layer accompanied by plasma formation and material redistribution. These processes determine not only the surface morphology but also the evolution of the subsurface region, where chemical gradients, defect structures, and mechanical properties are modified.

**Figure 4:**
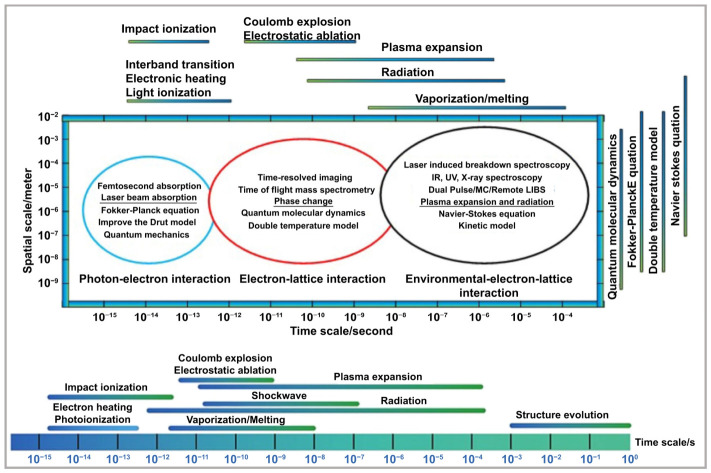
Illustration of a multiscale theoretical model of femtosecond laser–matter interaction. The scheme demonstrates the integration of fundamental physical phenomena (photoionization, Coulomb explosion, electron heating) with subsequent structural evolution of the material [95,96].

Because conventional thermal descriptions are insufficient for capturing such multiscale dynamics, comprehensive theoretical approaches based on multiphysics and multiscale modeling have been developed. Figure 4 illustrates a representative framework describing femtosecond laser–matter interaction across temporal and spatial scales [95,96]. Such models integrate ultrafast phenomena, including photoionization, interband transitions, electron heating, Coulomb explosion, and plasma expansion, with subsequent processes occurring on longer timescales, such as melting, shock-wave propagation, resolidification, and structural relaxation. Application of these approaches provides insight into how transient energy redistribution governs the final morphology, chemical reconstruction, and mechanical response of the laser-modified brass surface.

A direct analogy between femtosecond laser-induced ZnO formation on brass and plasma-assisted oxide synthesis can be established. In plasma-based ZnO production methods, including supersonic plasma jet deposition and plasma-enhanced atomic layer deposition, oxide formation is controlled by the interaction between reactive plasma species and the substrate, followed by nucleation and crystal growth processes [97,98]. Although conventional plasma deposition occurs under quasi-steady conditions, laser-induced plasma exists only transiently within the ablation zone; both approaches involve similar fundamental stages: generation of reactive species, transport toward the surface, oxide nucleation, and structural stabilization.

**Figure 5:**
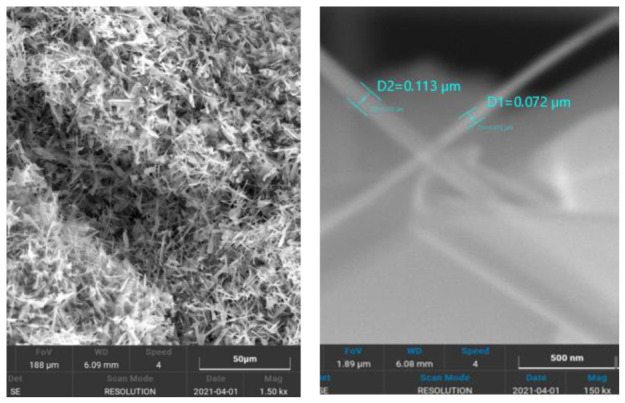
Zinc oxide nanostructures (1500× magnification) [99].

Comparison with plasma-assisted ZnO synthesis demonstrates that oxide morphology and functional properties are governed by the balance between energy input, concentration of active species, and growth kinetics. ZnO formation generally proceeds through successive stages of nucleation, growth, and crystallization. SEM observations of plasma-produced ZnO structures reveal a wide range of morphologies, including platelet-like and rod-like architectures, depending on synthesis conditions (Figure 5) [99]. In laser-processed brass, these processes occur under highly localized nonequilibrium conditions, where plasma generation is coupled with selective zinc redistribution from the alloy matrix and diffusion-driven transport within the near-surface region. Therefore, laser-induced ZnO formation can be considered a specific case of plasma-assisted oxide synthesis in which the reactive medium and the metal source are generated simultaneously.

**Figure 6:**
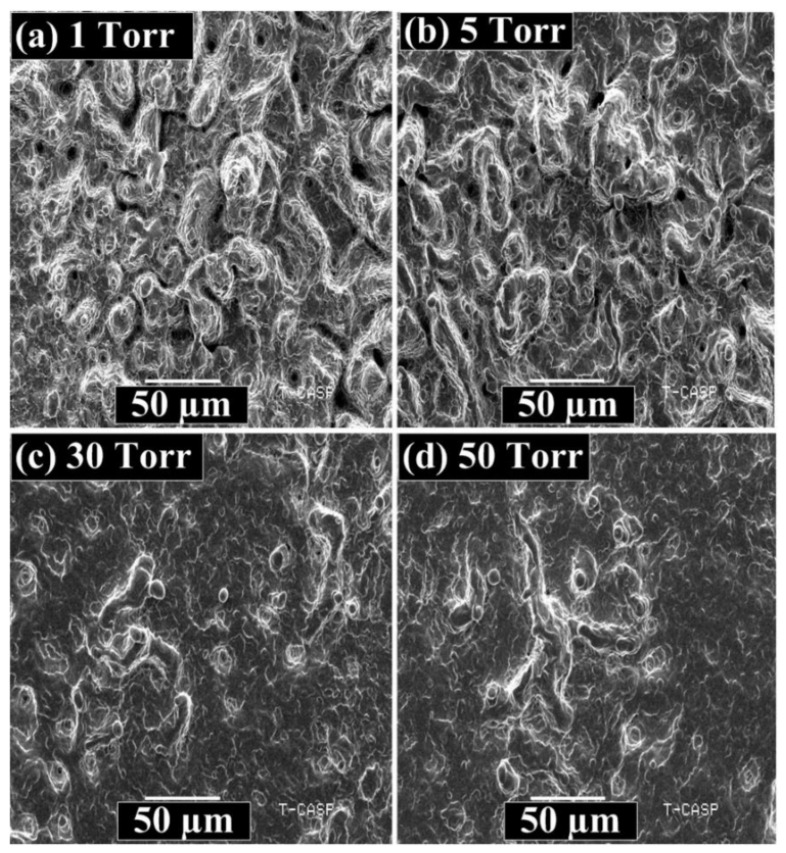
Surface morphology of Cu–Zn alloy after nanosecond laser ablation in argon atmosphere at different pressures: (**a**) 1 Torr, (**b**) 5 Torr, (**c**) 30 Torr, and (**d**) 50 Torr. Ridges, cavities, cone-like structures, melt droplets, and flow traces are observed [100].

The surrounding environment strongly affects the structural evolution of laser-treated Cu–Zn alloys. Although Ref. [100] investigated nanosecond laser ablation rather than femtosecond processing, the reported dependence of morphology on ambient pressure provides important insight into the role of plasma confinement and material transport. Changes in gas pressure modify plasma expansion dynamics, redeposition behavior, and melt flow, resulting in the formation of ridges, cavities, cones, droplets, and flow-related structures (Figure 6). These observations demonstrate that external conditions influence not only material removal but also the redistribution pathways of alloy components during laser irradiation.

Despite significant progress in understanding femtosecond laser interaction with brass, the relationship between oxidation, elemental redistribution, and structural modification remains complex. Classical descriptions of ultrafast ablation emphasize sub-thermal material removal, where energy is initially deposited into the electronic subsystem and transferred to the lattice over picosecond timescales. However, multicomponent alloys exhibit additional mechanisms, including selective component redistribution, oxide formation, and development of chemically graded regions [101].

**Figure 7:**
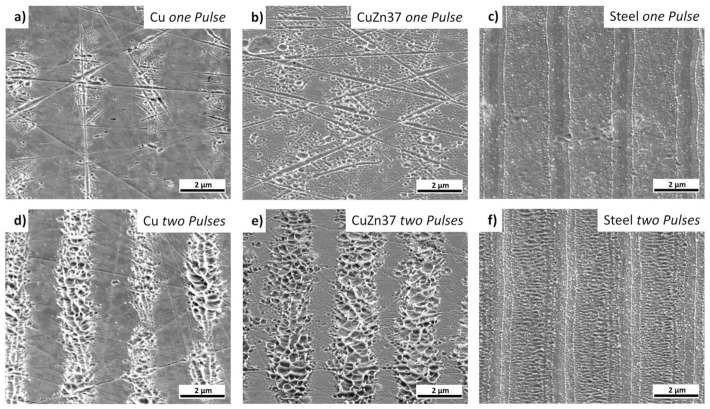
SEM images of periodic structures (period 3 μm) formed by USP-DLIP at a fluence of 1.1 J/cm^2^: (**a**,**d**) copper, (**b**,**e**) CuZn37, (**c**,**f**) stainless steel. (**a**–**c**) single pulse; (**d**–**f**) double pulse irradiation [101].

The influence of alloy composition becomes particularly evident during hierarchical structuring processes. DLIP experiments demonstrate that Cu and brass exhibit different ablation behavior under comparable irradiation conditions (Figure 7). The presence of zinc decreases the effective ablation threshold, resulting in differences in evaporation dynamics and processed-zone dimensions between pure copper and CuZn37 alloy. Furthermore, repeated irradiation modifies surface optical properties through the formation of nanoscale roughness and oxide-containing regions, which increase absorption and influence subsequent material removal.

The energy regime characteristic of femtosecond processing is defined by strong nonequilibrium between the electronic and lattice subsystems. During the pulse and early relaxation stages, the electronic subsystem reaches much higher effective temperatures than the lattice, controlling subsequent melting, ablation, and structural rearrangement. This nonequilibrium regime limits thermal diffusion and minimizes the heat-affected zone compared with longer pulse durations.

Under multipulse irradiation, cumulative effects become increasingly important. The progressive formation of LIPSS modifies local absorption conditions, enhancing the interaction of subsequent pulses with the already structured surface. Controlled variation in pulse energy sequences provides an additional mechanism for regulating self-organization processes and improving the regularity of periodic structures [102]. These observations demonstrate that the final morphology is determined not only by individual pulse parameters but also by the irradiation history and cumulative energy deposition.

Femtosecond laser processing also modifies the subsurface crystalline structure of brass. Under extremely high strain rates (~10^9^–10^10^ s^−1^), rearrangement of the dislocation subsystem may occur, accompanied by transformation from network-like defect configurations toward more ordered lamellar structures [103]. Such changes are associated with high local stresses and transient electron-pressure effects generated during nonequilibrium excitation. Simultaneously, modifications in vacancy and dislocation populations influence the mechanical response of the near-surface region, contributing to changes in microhardness and residual stress state.

An additional contribution to subsurface reconstruction arises from the dynamics of the laser-induced plasma plume. Expansion of the plasma cloud involves spatial redistribution of charged particles and possible plume-splitting effects associated with ambipolar electric fields generated by electron–ion separation [104]. These processes influence the transport of ablated species and contribute to the formation of chemically heterogeneous regions near the surface. Thus, plasma evolution is directly coupled with both chemical and structural modification of the alloy.

The formation of oxide-containing layers in brass can therefore be considered as the result of three interconnected mechanisms: plasma-assisted activation, diffusion-driven zinc redistribution, and chemical fixation of zinc by oxygen. The plasma component determines the initial nonequilibrium conditions through localized energy deposition and generation of reactive species, whereas diffusion controls zinc migration from the alloy interior toward the modified region. The chemical stage stabilizes the redistributed zinc in the form of ZnO or related oxide phases. The efficiency of these processes depends strongly on environmental conditions, including pressure, plasma confinement, and reactive species transport [105].

**Figure 8:**
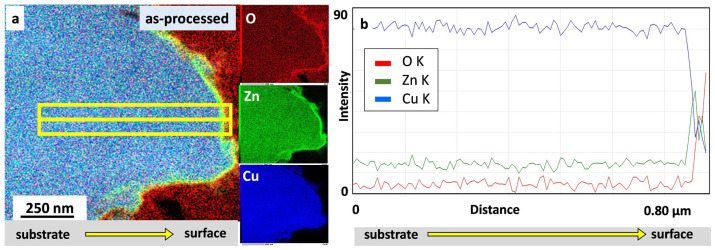
STEM-EDS mapping results of structured brass: (**a**) elemental distribution map in cross-section (from bulk to surface); (**b**) line scan intensity profile showing pronounced zinc segregation [106].

Experimental investigations confirm pronounced zinc migration toward the laser-affected region during femtosecond processing. STEM-EDS cross-sectional analysis of CuZn15 after laser treatment demonstrates the formation of a chemically modified near-surface layer with copper depletion and simultaneous enrichment in zinc and oxygen (Figure 8) [106]. The bulk composition remains comparatively unchanged, whereas the surface region exhibits a pronounced concentration gradient. The absence of strong crystalline oxide reflections in GI-XRD measurements, combined with elemental analysis, indicates that the oxide-containing layer may possess a predominantly amorphous or nanocrystalline structure, which is consistent with ultrafast cooling after irradiation.

The chemical nature of the modified layer has been further confirmed by selective chemical etching experiments. Reduction of oxygen concentration after treatment with citric acid indicates that oxygen is incorporated into a soluble oxide phase formed at the surface. These results demonstrate that laser-induced modification of brass cannot be explained solely by mechanical ablation. Instead, the process involves coupled zinc migration, oxidation reactions, and structural reconstruction of the near-surface region.

The resulting gradient architecture, characterized by copper depletion, zinc enrichment, oxide formation, and hierarchical surface morphology, determines the functional response of the modified alloy [107]. Selective oxidation of zinc generates concentration gradients that further promote zinc transport from the bulk toward the surface, creating a dynamic redistribution process [108]. Under femtosecond irradiation, these effects are enhanced by defect-assisted diffusion and transient increases in atomic mobility.

**Figure 9:**
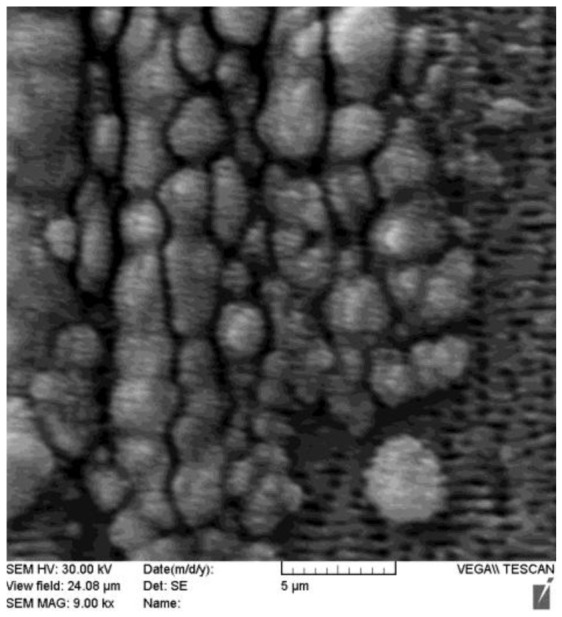
Evolution of a nanostructured Cu–Zn surface under multi-pulse femtosecond laser irradiation (below the ablation threshold): formation of large-scale zinc oxide structures (up to 3 μm) on the left with local contrast changes associated with compositional redistribution and surface oxidation alongside periodic ridge-like structures with a characteristic spacing of approximately 0.68 μm on the right [108].

Figure 9 illustrates the evolution of a nanostructured Cu–Zn surface during multipulse femtosecond irradiation below the ablation threshold. Sequential exposure results in the development of large-scale zinc oxide structures (up to 3 μm) with local contrast changes associated with compositional redistribution and surface oxidation, alongside periodic ridge-like structures with characteristic spacing of approximately 0.68 μm [108]. These observations demonstrate that the subsurface region remains actively involved in structural evolution even after the initial laser pulse.

The cumulative effect of repeated irradiation leads to the formation of a persistent nonequilibrium near-surface state. Incomplete relaxation between successive pulses maintains elevated defect activity and enhances diffusion-driven transport. Although the duration of an individual femtosecond pulse is extremely short, post-pulse processes strongly influence the final material state because localized energy accumulation preserves atomic mobility for times exceeding the initial excitation period [109]. As a result, zinc redistribution and oxide formation continue during relaxation, contributing to the development of an extended ZnO-containing surface layer.

SEM investigations of CuZn37 after laser treatment further confirm the presence of oxidation products and redeposited particles within the structured region [110]. These features indicate that laser processing induces continuous chemical and structural evolution of the interaction zone rather than producing a static modified surface. The near-surface layer therefore represents an active region where plasma transport, diffusion, oxidation, and morphological self-organization occur simultaneously.

Comparison between different pulse-duration regimes highlights the unique role of femtosecond irradiation. Picosecond and nanosecond pulses involve stronger lattice heating, thermomechanical stresses, and plasma shielding effects, resulting in more pronounced thermal modification and nonlinear ablation behavior [111]. In contrast, femtosecond processing is dominated by ultrafast electron–phonon dynamics, enabling precise control over surface reconstruction while limiting extensive thermal damage.

The hierarchical combination of micro- and nanoscale structures produced by DLIP and LIPSS provides additional control over the functional properties of brass surfaces. Such architectures modify optical response, wettability, interfacial transport, and mechanical behavior [112,113]. When combined with chemical modification through zinc oxidation, laser structuring transforms brass from a passive alloy into a tunable functional material system.

Changes in mechanical properties are closely related to the formation of oxide-containing hardened surface layers and modifications of the defect structure. Increased microhardness has been associated with the formation of rigid ZnO-containing frameworks and rearrangement of dislocation structures [114]. However, excessive energy input may lead to structural degradation, increased defects, and reduced mechanical performance. Therefore, optimization of laser parameters requires balancing oxide formation, structural strengthening, and preservation of the metallic matrix.

For quantitative analysis of chemically heterogeneous surfaces, advanced spatially resolved characterization methods are required. The combination of femtosecond laser ablation–spark-induced breakdown spectroscopy with machine-learning approaches enables accurate mapping of elemental distributions and identification of laser-induced compositional gradients [115]. Such approaches are particularly valuable for separating plasma-driven redistribution from diffusion-controlled processes during ZnO formation.

Overall, femtosecond laser modification of brass represents a multilevel chemo-mechanical transformation process involving electronic excitation, plasma evolution, zinc redistribution, oxidation, defect restructuring, and morphological self-organization. The final properties of the modified surface are determined by the interaction of these mechanisms across multiple spatial and temporal scales. This coupled chemo-morphological evolution defines the potential of femtosecond laser processing as an advanced strategy for engineering functional ZnO-containing surfaces on industrial Cu–Zn alloys.

## 7. Discussion

The evidence discussed in this review indicates that femtosecond laser modification of brass should be considered as a multilevel nonequilibrium process involving the coupled evolution of chemical composition, surface morphology, plasma dynamics, and interfacial reactions [12,16,31,95,106]. The conceptual framework developed here integrates direct experimental observations obtained for Cu–Zn alloys with established concepts of ultrafast laser–matter interaction. In particular, selective Zn redistribution, formation of Zn-enriched near-surface regions, and laser-induced chemical modification have been directly observed in Cu–Zn systems, whereas the interpretation of ultrafast energy transfer, plasma-assisted oxidation pathways, and nonequilibrium reaction mechanisms is supported by broader studies of femtosecond laser processing and plasma-assisted oxide formation.

Unlike conventional descriptions that treat electron–lattice relaxation, ablation, oxidation, and surface functionalization as partially independent phenomena [13,21,22,37], the present analysis considers them as interconnected stages of a single laser-induced transformation process. This approach does not introduce a new physical model but provides an integrated interpretation of available experimental and theoretical results, emphasizing the coupling between nonequilibrium electronic excitation, Zn redistribution, plasma evolution, heterogeneous oxidation, and morphological self-organization [31,35,104,106,109]. Within this framework, laser-induced ZnO formation is regarded as the result of coupled mass transport, plasma-assisted reactions, and surface reconstruction rather than as an isolated oxidation event.

A key conclusion emerging from the reviewed studies is that zinc plays an active kinetic role in the evolution of laser-modified brass. In conventional applications, Zn mobility in Cu–Zn alloys is frequently associated with undesirable phenomena such as dezincification [1]. However, under femtosecond laser irradiation, this intrinsic mobility becomes a mechanism that enables controlled chemical modification. Under nonequilibrium laser-induced conditions, the enhanced mobility of Zn relative to Cu promotes selective redistribution and the development of Zn-enriched regions within the laser-affected zone [28]. Experimental studies of laser-treated brass confirm the formation of modified near-surface layers with altered elemental composition, including zinc enrichment and oxygen incorporation [106,109]. These regions provide favorable conditions for subsequent oxidation and ZnO nucleation. Therefore, Zn redistribution should be considered not merely as a consequence of laser-induced heating, but as an initial chemical conditioning stage that influences the subsequent formation and stabilization of oxide-containing surface layers.

The reviewed literature further demonstrates that plasma evolution and solid-state mass transport are strongly coupled during femtosecond laser processing [22,32,104]. The laser-induced plasma acts simultaneously as a source of reactive species, a transport medium, and a transient reaction environment. Within the expanding plume, ionization, dissociation of ambient molecules, generation of oxygen-containing reactive species, oxidation of Zn-containing vapor species, and condensation of oxide-containing nanoparticles may occur concurrently [31,62,63,105]. At the same time, zinc redistribution within the alloy continuously modifies the chemical composition of the interaction zone. Consequently, ZnO formation is likely governed by a combination of plume-mediated processes and heterogeneous reactions occurring at the alloy–plasma interface, including oxygen adsorption, oxidation of zinc-enriched regions, nucleation, and subsequent oxide growth [69,71,80].

An important aspect of this mechanism is that ZnO formation cannot be assigned exclusively to either gas-phase reactions or surface oxidation. The plasma plume contributes to the generation and transport of reactive oxygen-containing species, whereas the alloy surface provides the zinc source and the structural environment required for oxide stabilization. The interaction between these two subsystems creates a transient reaction zone characterized by high chemical activity, local compositional gradients, and strong deviations from equilibrium conditions. Such a description is consistent with plasma-assisted ZnO synthesis approaches, where oxide formation depends on the balance between reactive species generation, transport, and incorporation into the growing phase [64,66,67,68,74].

Surface morphology represents an additional controlling factor in this coupled process. LIPSS and DLIP are not simply passive products of material removal but actively modify subsequent laser–matter interaction [15,54]. Periodic and hierarchical structures influence local electromagnetic-field distribution, absorption behavior, thermal transport, and mass-transfer pathways [18,48,52]. As a result, morphology can modify local reaction conditions and create preferential sites for ZnO nucleation within the laser-modified region [36,106,109]. This establishes a feedback relationship in which chemical modification influences morphology, while the evolving morphology further affects subsequent energy deposition and reaction kinetics.

Accordingly, ZnO functionalization of brass should be interpreted as the result of multiple competing and cooperative mechanisms operating across different temporal and spatial scales. Depending on laser fluence, pulse accumulation, scanning conditions, and environmental parameters, the system may evolve toward predominantly morphological modification or toward more pronounced chemical reconstruction accompanied by oxide formation and stabilization [10,19,42,81]. The final surface state therefore represents a balance between material removal, zinc transport, oxidation kinetics, and structural self-organization.

Comparison with plasma-assisted and gas-phase ZnO synthesis methods further supports this interpretation. Although conventional plasma deposition and femtosecond laser processing differ significantly in duration, geometry, and energy delivery, both involve a similar sequence of fundamental stages: generation of reactive species, transport, heterogeneous reaction, nucleation, and crystal growth [64,70]. The distinctive feature of femtosecond laser processing is that these stages occur within a highly localized and transient environment, where the reactive medium, zinc source, and modified substrate are generated simultaneously during a single processing event [11,30,76].

**Figure 10:**
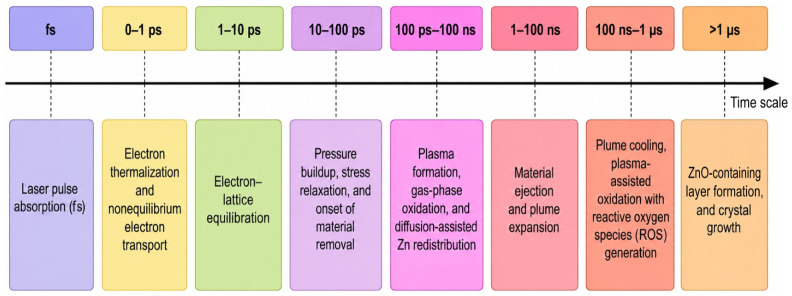
Multiscale temporal sequence of physical and chemical processes during femtosecond laser processing of Cu–Zn alloys, leading from ultrafast energy absorption to ZnO-containing layer formation: laser pulse absorption (fs) [12,21,22]; electron thermalization and nonequilibrium electron transport [13,27]; electron–lattice equilibration [23,24,25,26]; pressure buildup, stress relaxation, and onset of material removal [21,22,29]; plasma formation, gas-phase oxidation, and diffusion-assisted Zn redistribution [28,31,34,100,105,106,109]; material ejection and plume expansion [31,34,95,96,104]; plume cooling, plasma-assisted oxidation with reactive oxygen species (ROS) generation [32,62,63,64,95,96,100,104,105]; and ZnO-containing layer formation and crystal growth (including DLIP structuring) [35,36,69,71,97,98,106,109]. The temporal ranges represent characteristic values synthesized from the cited literature.

Figure 10 summarizes the characteristic temporal dynamics of the coupled physical and chemical processes involved in femtosecond laser modification of Cu–Zn alloys, with the corresponding spatial scales systematized throughout this discussion. The initial stage consists of ultrafast energy absorption and electronic excitation occurring within femtoseconds [12,21,22]. During the following picoseconds, electron thermalization, nonequilibrium electron transport, and electron–lattice equilibration govern energy redistribution within the material [13,23,24,25,26,27]. After electron–lattice equilibration, rapid pressure buildup, stress relaxation, and the onset of material removal dominate the subsequent evolution over picosecond to sub-nanosecond timescales [21,22,29]. At longer timescales (approximately 100 ps–100 ns), plasma formation and expansion, gas-phase reactions, nanoparticle generation, diffusion-assisted Zn redistribution, and nanoscale phase modification become increasingly important [31,32,34,100,105]. Subsequent plasma cooling, reactive species transport, oxide nucleation, and crystal growth extend into the nanosecond-to-microsecond range and beyond [35,36,62,63,64,69,71,97,98].

The characteristic spatial scales of these processes span several orders of magnitude and can be categorized into two distinct zones. Within the solid alloy, nanoscale phenomena occur near the surface, including optical absorption (approx. 10–20 nm), nonequilibrium electron transport (approx. 50–100 nm), and the formation of a Zn-enriched subsurface layer (several tens of nanometers). Conversely, gas-phase processes above the irradiated surface operate on a microscale, with the expanding plasma plume reaching heights of several hundred micrometers [21,22,28,32,104,106,109]. These values should be regarded as representative rather than universal, since the exact evolution depends on laser parameters, alloy composition, and processing atmosphere.

**Figure 11:**
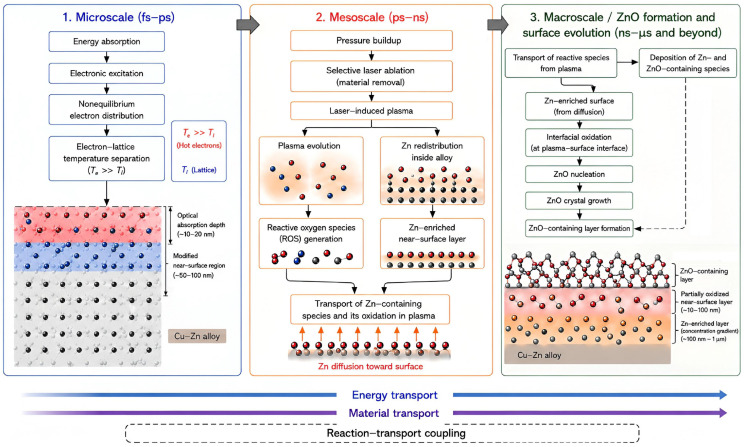
Conceptual multiscale framework for interpreting ZnO formation on Cu–Zn alloys during femtosecond laser processing.

While Figure 10 focuses on the temporal dynamics of the individual processes, Figure 11 illustrates their physicochemical coupling. The proposed reaction–transport framework integrates nonequilibrium electronic excitation, plasma evolution, diffusion-assisted Zn redistribution, heterogeneous oxidation, and morphological evolution into a unified description of ZnO-containing layer formation. This interpretation is supported by tracer diffusion studies of Cu–Zn alloys [28], compositional characterization of laser-modified brass [106,109], investigations of laser-induced plasma chemistry and nanoparticle formation [31,100,105], studies of reactive oxygen species generation [62,63,64], and investigations of ZnO nucleation and growth mechanisms [35,36,69,71,97,98].

Within this framework, nonequilibrium laser excitation generates temperature and chemical-potential gradients that promote preferential Zn redistribution inside the alloy [28,106,109]. Simultaneously, plasma formation produces reactive oxygen-containing species and provides transport pathways for Zn-containing products above the irradiated surface [31,62,63,64,104,105]. The interaction of these pathways promotes heterogeneous oxidation at the plasma–surface interface, where oxygen-containing species interact with Zn-enriched regions, creating favorable conditions for ZnO nucleation and subsequent oxide growth within the modified surface layer [35,36,69,71,97,98]. Thus, ZnO-containing layer formation is best understood as a coupled reaction–transport phenomenon involving diffusion, plasma chemistry, interfacial oxidation, and morphology-dependent feedback.

Taken together, the reviewed evidence supports the interpretation of femtosecond laser processing of Cu–Zn alloys as an open nonequilibrium system in which energy transport, elemental redistribution, plasma evolution, chemical reactions, and structural reconstruction proceed simultaneously across multiple scales. The resulting ZnO/Cu–Zn transition zone emerges from the collective evolution of these coupled processes rather than from a single dominant mechanism. This integrated view provides a consistent basis for understanding laser-induced oxide functionalization and for designing ZnO-containing surface architectures on industrial brass alloys through controlled femtosecond laser processing.

Despite substantial progress, several important challenges remain unresolved. Quantitative relationships between ultrafast laser excitation, Zn redistribution kinetics, and subsequent ZnO formation have not yet been fully established through combined experimental and computational approaches. Direct characterization of the transient plasma–surface interface, including generation, transport, and incorporation of oxygen-containing reactive species, remains difficult at ultrafast timescales. Furthermore, although laser-induced architectures such as LIPSS and DLIP clearly influence local transport and reaction conditions, the mechanisms linking surface morphology with oxide nucleation and growth require additional investigation. Future advances will likely depend on combining time-resolved diagnostics, multiscale modeling, and systematic processing–structure–property correlations. Such developments will enable a transition from empirical optimization toward predictive design of functional laser-modified brass surfaces.

## 8. Conclusions

This review establishes an integrated multiscale physico-chemical framework for understanding the surface engineering of Cu–Zn alloys through femtosecond laser processing. The analysis of theoretical and experimental studies demonstrates that laser-induced modification is governed by the coupled evolution of nonequilibrium electronic excitation, plasma formation, atomic redistribution, and interfacial chemical reactions. These interconnected processes result in selective zinc redistribution, generation of a reactive plasma environment, reaction–transport interactions, and formation of ZnO-containing surface layers under strongly nonequilibrium conditions.

During femtosecond laser irradiation, zinc redistribution plays a central role in determining the chemical evolution of the Cu–Zn surface. The enhanced mobility of Zn under transient laser-induced conditions promotes the development of Zn-enriched near-surface regions, which subsequently facilitate oxidation and the formation of ZnO-containing surface structures. At the same time, the laser-induced plasma plays multiple roles: it generates reactive oxygen-containing species, facilitates their transport toward the modified surface, and contributes to the redistribution of ablated and condensed species within the interaction zone. In parallel, morphological self-organization through LIPSS and DLIP formation modifies local electromagnetic-field distribution, energy absorption, and mass-transport pathways, establishing feedback between surface architecture and chemical transformation.

Comparison with plasma-assisted and gas-phase ZnO synthesis approaches shows that oxide formation follows a general sequence involving generation of reactive species, transport, interfacial reactions, nucleation, and crystal growth. The specific feature of femtosecond laser processing is that these stages occur within a transient and spatially confined reaction environment generated directly from the Cu–Zn substrate. As a result, material redistribution, surface structuring, and oxide formation proceed simultaneously during a single processing event. Laser-induced ZnO functionalization can therefore be considered as the result of coupled ultrafast energy deposition, plasma chemistry, diffusion-controlled transport, heterogeneous oxidation, and morphology evolution.

Further development of this field requires quantitative descriptions of the relationships between laser parameters, transient mass transport, plasma chemistry, and oxide-growth kinetics. Progress will depend on combining time-resolved experimental diagnostics with multiscale computational approaches and systematic processing–structure–property correlations. Such efforts will provide a pathway toward predictive control of ZnO-containing functional layers and the rational design of laser-engineered surfaces on Cu–Zn alloys.

## Data Availability

The data presented in this study are available in this article.
